# The origin and the genetic regulation of the self-compatibility mechanism in clementine (*Citrus clementina* Hort. ex Tan.)

**DOI:** 10.3389/fpls.2024.1360087

**Published:** 2024-03-04

**Authors:** Stefania Bennici, Lara Poles, Mario Di Guardo, Lawrence Percival-Alwyn, Mario Caccamo, Concetta Licciardello, Alessandra Gentile, Gaetano Distefano, Stefano La Malfa

**Affiliations:** ^1^ Department of Agriculture, Food and Environment (Di3A), University of Catania, Catania, Italy; ^2^ National Institute of Agricultural Botany (NIAB), Cambridge, United Kingdom; ^3^ Council for Agricultural Research and Economics (CREA) - Research Centre for Olive, Fruit and Citrus Crops, Acireale, Italy

**Keywords:** ‘Monreal’ genome assembly, RNA-Seq, SNPs discovery, S-RNase, S-genotyping, seedlessness

## Abstract

Self-incompatibility (SI) is a genetic mechanism common in flowering plants to prevent self-fertilization. Among citrus species, several pummelo, mandarin, and mandarin-like accessions show SI behavior. In these species, SI is coupled with a variable degree of parthenocarpy ensuring the production of seedless fruits, a trait that is highly appreciated by consumers. In *Citrus*, recent evidences have shown the presence of a gametophytic SI system based on *S-ribonucleases* (*S-RNases*) ability to impair self-pollen tube growth in the upper/middle part of the style. In the present study, we combined PCR analysis and next-generation sequencing technologies, to define the presence of *S_7_-* and *S_11_-Rnases* in the *S*-genotype of the *Citrus clementina* (Hort. ex Tan.), the self-incompatible ‘Comune’ clementine and its self-compatible natural mutant ‘Monreal’. The reference genome of ‘Monreal’ clementine is presented for the first time, providing more robust results on the genetic sequence of the newly discovered *S_7_-RNase.* SNP discovery analysis coupled with the annotation of the variants detected enabled the identification of 7,781 SNPs effecting 5,661 genes in ‘Monreal’ compared to the reference genome of *C. clementina*. Transcriptome analysis of unpollinated pistils at the mature stage from both clementine genotypes revealed the lack of expression of *S_7_-RNase* in ‘Monreal’ suggesting its involvement in the loss of the SI response. RNA-seq analysis followed by gene ontology studies enabled the identification of 2,680 differentially expressed genes (DEGs), a significant number of those is involved in oxidoreductase and transmembrane transport activity. Merging of DNA sequencing and RNA data led to the identification of 164 DEGs characterized by the presence of at least one SNP predicted to induce mutations with a high effect on their amino acid sequence. Among them, four candidate genes referring to two *Agamous*-like MADS-box proteins, to *MYB111* and to *MLO*-like protein 12 were validated. Moreover, the transcription factor *MYB111* appeared to contain a binding site for the 2.0-kb upstream sequences of the *S_7_-* and *S_11_-RNase* genes. These results provide useful information about the genetic bases of SI indicating that SNPs present in their sequence could be responsible for the differential expression and the regulation of *S_7_-RNase* and consequently of the SI mechanism.

## 1 Introduction

Self-incompatibility (SI) is a genetic mechanism that prevents self-fertilization in flowering plants. The mechanism plays a crucial role in promoting outcrossing and, as a consequence, increases the genetic variability within a species. SI in citrus is related to seed formation and fruit set which has been mainly described in pummelo (*Citrus maxima* (Burm.) Merr., 1917) and mandarin (*C. reticulata* Blanco, 1837), even though this mechanism is also relevant in mandarin-like varieties such as clementine and several hybrids (Yamamoto et al., 2006). Seedlessness is one of the most consumer desirable fruit quality traits. In the most economically important citrus species, SI, in conjunction with parthenocarpy, plays a crucial role in producing seedless fruits when plants are cultivated in solid blocks to prevent cross-pollination (Distefano et al., 2009b; Montalt et al., 2021). Even though, the latter is the most widely diffused, other mechanisms leading to seedlessness in parthenocarpic citrus genotypes have been described, such as: female and/or male sterility (Goto et al., 2016; Catalano et al., 2022), ovule/embryo degeneration (Mesejo et al., 2014), irradiation (Goldenberg et al., 2014), triploidy (Ollitrault et al., 2007) or temperature stress conditions (Bennici et al., 2019; Aloisi et al., 2020; Montalt et al., 2022).

Clementine (*C. clementina* Hort. ex Tan.) is the most representative SI species within the mandarin group (Ngo et al., 2001; Chao, 2005; Distefano et al., 2009a; Honsho, 2023). Clementine was selected from a mandarin chance seedling and now represents the principal mandarin varietal group cultivated in the Mediterranean basin. As a result of spontaneous bud sport mutations from the original ‘Comune’ clementine, many different clementine varieties have been selected. In addition, several cultivars have been originated by crossing the SI clementine with other varieties or hybrids (Montalt et al., 2021; Honsho, 2023). Some reports described cross-incompatibility in sibling cultivars obtained from crosses using clementine as the female (Kitajima et al., 2001) or male (Yamamoto et al., 2006) parent.

The SI system is controlled by a single genomic polymorphic region, the *S-locus*, that contains two tightly linked genes: the pollen and pistil determinants. Both are multi-allelic, and their interaction is responsible for the self and inter-compatibility or incompatibility within a species. Among the described SI systems, the gametophytic SI (GSI) based on the *S-RNase* is the most widespread and is found in several species belonging to the families of *Solanaceae*, *Rosaceae*, *Plantaginaceae* and *Rutaceae*. According to this model, the female *S* determinant, encoded by a class of III S ribonuclease (*S-RNase*), abundantly present in style tissues, penetrates the ‘self’ pollen tube and inhibits its growth by degrading RNAs (McClure et al., 2011). The male *S* determinant comprises multiple *S-locus F-boxes* (SLFs) that are the component of a SKp1-Culling-F-box (SCF) complex promoting the growth of compatible pollen by ubiquitinating and degrading non-self *S-RNases* in a 26A proteasome-dependent manner (Hu et al., 2021). To date, eighteen *S-RNases*, seventeen SI and one SC, have been reported in pummelo (*S_1_-S_9_, S_16_*), mandarin (*S_10_-S_11_*), sweet orange (*C*. *sinensis* (L.) Osbeck; *S_m_*) and in other citrus species (*S_12_-S_15_, S_17_*) (Honsho, 2023). The citrus *S-RNases* are characterized by the same general structure as those found in *Plantaginaceae*, *Solanaceae*, and *Rosaceae*, all showing five conserved domains (C1–C5) with three histidine residues and 5 hypervariable domains (HV1–HV5) with a single intron within the HV1 (Liang et al., 2020; Honsho et al., 2021). Furthermore, a *S-RNase* (*S_m_-RNase*) characterized by a mutation causing the loss of SI was isolated in sweet orange (Liang et al., 2020). Genomic analysis supports the origin of the *S_m_-RNase* from wild mandarin as a spontaneous mutation, that was eventually transmitted to its hybrids and fixed (Liang et al., 2020).

Despite its significant economic importance, the molecular basis of SI in the most widely cultivated *Citrus* species remains not fully elucidated. Among the *Citrus* species already reported, ‘Monreal’ clementine represents a SC natural mutant of the SI clementine (Distefano et al., 2009a). The Japanase cultivars ‘Ihara Hyuga’, ‘Nishiuchi Konatsu’, ‘Shiratori Hyuga’, and an unregistered line, are SC mutants of ‘Hyuganatsu’ (*C. tamurana* hort. ex Tan.; SI) (Honsho, 2023); while the Chinese cultivars ‘Guiyou No. 1’ and ‘Zigui Shatian’ are SC mutants of ‘Shatian’ pummelo (SI; Honsho, 2023). Lack of SI has been associated with attenuated or abolished expression of the *S-RNase* in Japanese citrus cultivars (Honsho et al., 2021), sweet orange (Liang et al., 2020) and Chinese varieties of pummelo (Hu et al., 2021). In sweet orange downregulation of *S-RNase* (*S_m_*) has been associated with a single nucleotide deletion affecting the *S-RNase* CDS responsible for a premature stop codon (Liang et al., 2020); while in an SC mutant of pummelo was associated with a candidate gene (*CgHB40*) that potentially contributes to the transcriptional regulation of *S-RNase* promoters (Hu et al., 2021).

In this context, clementine is often characterized by SI, even though natural SC mutants exist. Distefano et al. (2009a) performed the self and reciprocal crosses of the SI ‘Comune’ clementine and its SC mutant ‘Monreal’ demonstrating that the mutation leading to SC affected the pistil functions. This finding was demonstrated by histological analyses of the pollen tubes growth that did not show anomalies in ‘Monreal’ (both self- and cross-pollinated). Whereas, in the self- and cross-pollinated ‘Comune’, the pollen tubes arrested their growth in the upper or middle style (thus recognizing the pollen of the SC mutant as self-pollen activating the SI reaction).

In this work, the same two clementine varieties, ‘Comune’ (ComSI) and ‘Monreal’ (MonSC) were analyzed to characterize the genetic bases of the SI mechanism through whole-genome DNA and RNA sequencing approaches. The combination of the PCR analysis based on the known *S*-alleles and the *de novo* sequencing of the ‘Monreal’ genome enabled the identification, for the first time, of the complete *S*-genotype of clementine and assesses the mutations rate in MonSC compared to the *C. clementina* reference genome. In addition, the RNA-seq analysis of the pistil tissue and the downstream analysis of the polymorphisms at the coding and at the *S-RNase* promoter regions were combined to identify candidate genes associated with SI. The findings of this study provide novel insights on the genetic regulation of both the *S*-alleles and other genes related to the progamic phase, providing altogether novel hypothesis for the SI regulation mechanism in clementine.

## 2 Material and methods

### 2.1 Genomic analysis

#### 2.1.1 DNA extraction

DNA of both ComSI and MonSC was extracted from fresh young leaves collected from 10-year-old trees grown at the experimental field of the University of Catania (Italy). Genomic DNA was extracted using ISOLATE II Plant DNA Kits (Bioline, Meridian Life Science, Memphis, TN, USA) according to the manufacturer’s instructions. The extracted DNA was quality-checked using a Nanodrop 2000 spectrophotometer (Thermo Scientific, Waltham, MA, USA) and agarose gel electrophoresis.

#### 2.1.2 *Consensus* primer PCR amplification and Sanger sequencing

For the amplification of the genomic DNA corresponding to the *S-RNase* genes in citrus, *consensus* degenerate primers and allele-specific primers were designed (**Supplementary Table 1**) using the online Primer3 software (https://www.primer3plus.com) based on the alignment of *S-RNase* nucleotide sequences reported in the literature (**Supplementary Figure 1**). The C2 and C3 conserved regions were used for the design of the forward and reverse consensus primers, respectively (**Supplementary Table 1**).

PCR amplifications were performed in a total volume of 20 μL containing 100 ng genomic DNA, 1x PCR buffer II, 2 mM magnesium chloride, 0.2 mM dNTPs, 0.2 μM of each primer and 1 U of MyTaq DNA polymerase (Bioline). *Consensus* PCR amplifications were conducted in thermal cyclers Mastercycler Nexus Gradient (Eppendorf, Hamburg, Germany) under the following conditions: 94°C for 2 min, 15 cycles of 94°C for 1 min, 52°C for 1 min and 2 min at 72°C followed by 20 cycles of 94°C for 1 min, 53°C for 1 min and 2min at 72°C, then final elongation at 72°C for 30 min. Amplicons were separated by electrophoresis on 2% agarose gel and amplified bands, two for each genotype, were extracted and purified with ISOLATE II PCR and Gel Kit (Bioline) following manufacturer’s instructions. Purified bands were then Sanger-sequenced using Eurofins Genomics Tube Sequencing service (Eurofins Genomics Europe Sequencing GmbH). The Sanger sequences were blasted against the NCBI database and specific primer pairs were designed based on the HV regions of the *S_7_* and *S_11_-RNases* sequences reported in the literature (**Supplementary Figure 1**). The HV1 and HV5 regions were used for the design of the forward and reverse *S_7_-RNase* specific primers, while the HV1 and HV4 regions were used for the design of the forward and reverse *S_11_-RNase* specific primers, respectively (**Supplementary Table 1**). *S*-allele-specific PCR amplifications were conducted in both ComSI and MonSC using an initial denaturation step at 94°C for 10 min, followed by 35 cycles at 94°C for 30 sec, 57°C for 45 sec and 72°C for 2 min with a final elongation at 72°C for 10 min (primers listed in **Supplementary Table 1**). Amplicons were purified and sequenced as described above to evaluate the presence of mutations with respect to sequences deposited in the NCBI database and alignment against the already available *C. clementina* v.1 (NCBI RefSeq assembly: GCF_000493195.1). Sanger sequencing of the 1.5-kb upstream region of *S_7_-RNases* in both ComSI and MonSC were performed using specific primers (**Supplementary Table 1**) using the amplification and analysis methods described above.

#### 2.1.3 ‘Monreal’ clementine sequencing and genome assembly

*De novo* assembly of the MonSC genome was performed by combining short and long reads technologies. Short read sequencing was performed using Illumina next-generation technology (PE-150 reads) which resulted an average read depth of 100X (30.13 Gb), while long reads sequencing was carried out using Oxford Nanopore Technology (ONT) with an average read depth of 30X (9.03 Gb). The genome assembly was based on a two-step approach: first, the ONT reads were assembled using the Flye aligner (Kolmogorov et al., 2019), then the overall quality of the draft assembly was improved by integrating the Illumina reads using the Pilon software (Walker et al., 2014). The completeness of the *de novo* assembly was tested with the Benchmarking Universal Single-Copy Orthologs (BUSCO v 5.4.3) (Simão et al., 2015; Manni et al., 2021) using the ‘embryophyta’ lineage (-l parameter) featuring 1,614 target genes.

#### 2.1.4 Identification of SNPs between ComSI and MonSC

The identification of SNPs occurring between ComSI and MonSC mandarins was performed using SnpEff software (Cingolani et al., 2012). The MonSC Illumina reads were aligned against the annotated *C. clementina* v.1 reference genome (NCBI RefSeq assembly: GCF_000493195.1) (Wu et al., 2014) using the Burrows-Wheeler Aligner (BWA) software package (Li and Durbin, 2009) The analysis allowed the identification of SNPs located within genes or coding sequences. SNP distribution along MonSC chromosomes was displayed employing the R package chromoMap (Anand and Rodriguez Lopez, 2022).

### 2.2 Transcriptome analysis

#### 2.2.1 RNA extraction

Total RNA was extracted from frozen virgin styles and stigmas of ComSI and MonSC collected 24h after anthesis using the Spectrum™ Plant Total RNA Kit (Sigma-Aldrich, Saint Louis, USA) and treated with DNase I (On-Column DNase I Digestion Set, Sigma-Aldrich, Saint Louis, USA) according to the manufacturer’s instructions. Extracted RNA was quantified using a NanoDrop-2000 (Thermo Scientific, USA) spectrophotometer and total RNA integrity was assayed using 1% agarose gel electrophoresis and then stored at −80°C for further analysis.

#### 2.2.2 RNA-seq analysis

Three biological replicates for each accession were employed for RNA-seq analysis (Illumina 150PE reads). Total RNA extracted from ComSI and MonSC was employed for library preparation and sequencing (provided by the Novogene company, Beijing, China). Raw reads (with an average of 20 million reads per sample) were aligned against the reference genome employing the Spliced Transcripts Alignment to a (STAR) RNA-seq aligner (Dobin et al., 2013); then reads were counted using the FeatureCounts software (Liao et al., 2014) Differentially expressed genes (DEGs) were identified using the DESeq2 R package (Love et al., 2014; Ignatiadis et al., 2016) significant DEGs were determined using a FoldChange ≥ 2 and an adjusted *p*-value ≤ 0.01. Gene Ontology (GO) and Kyoto Encyclopedia of Genes and Genomes (KEGG) databases enrichment analysis of DEGs were performed using the ShinyGO bioinformatic tool (Ge et al., 2020).

### 2.3 Selection of candidate genes based on DEGs, SNPs and *S-RNases* promoter analysis

By merging the results of DNA and RNA sequencing, candidate genes were selected for validation by quantitative real-time PCR (qRT-PCR). The conditions used for the selection of the candidate genes include DEGs: (i) affected by SNPs predicted to induce mutations with a high effect on their amino acid sequence; (ii) showing a function predicted by bibliographical analysis to be associated with the control of flowering and SI. Also, the 2.0-kb upstream sequences of *S_7_*- and *S_11_-RNase* genes were selected for the analysis of the *cis*-regulatory elements (CREs) using PlantCARE (https://bioinformatics.psb.ugent.be/webtools/plantare/html/) to identify motifs associated with candidate genes fulfilling the criteria described above.

### 2.4 Validation of candidate genes by qRT-PCR

*S_7_-RNase* and *S11-RNase* genes and a total of four candidate genes (*CICLE_v10023354mg, CICLE_v10006615mg, CICLE_v10015069mg and CICLE_v10005376mg*) were selected for qRT-PCR to verify differences in expression. Total RNA was extracted as described above from frozen virgin styles and stigmas of ComSI and MonSC collected at anthesis 24 h and 48 h after anthesis. The cDNA was synthesized from 1 μg of total RNA using the High-Capacity cDNA Reverse Transcription Kit (Applied Biosystems, Waltham, USA) according to the manufacturer’s instructions. qRT-PCR assays were run on the Rotor-Gene Q thermocycler (Qiagen, Hilden, Germany) in 20 μL total reaction volumes containing 1 × PCR buffer II, 2 mM MgCl2, 0.2 mM dNTPs, 0.3 μM of forward and reverse primer (Eurofins Genomics), 1.5 μM SYTO9 (Life Technologies, UK), 1 μL of the synthesized cDNA and 1 U of MyTaq DNA polymerase (Bioline, UK). Primers used for qRT-PCR analysis of candidate genes are shown in **Supplementary Table 1**. The citrus *Elongation Factor 1-alpha* gene (*EF-1α*, accession AY498567) was used as a housekeeping reference gene (Distefano et al., 2009a). Thermal cycling conditions included an initial denaturation at 95°C for 5 min, followed by 35 cycles at 95°C for 5 s, 59°C for 20 s, and 72°C for 2 min. The expression level of candidate genes relative to the EF-1α transcript was calculated following the mathematical model described by Livak and Schmittgen (2001). The values reported are the mean ± SD of at least three independent assays. Statistical analyses were performed using ANOVA (LSD test, *p* < 0.01).

## 3 Results

### 3.1 Genomic *S-RNase* identification in ComSI and MonSC using *consensus* primer PCR amplification and Sanger sequencing

The discovery of the *S*-genotypes of ComSI and MonSC was first carried out by employing *consensus* primers designed on the C2 and C3 conserved domains of the *S-RNase* (**Supplementary Table 1**; **Supplementary Figures 1, 2A**). Two bands of 284-bp and 260-bp were amplified in both samples (**Supplementary Figure 2B**). The sequencing of the PCR band of 284-bp showed a perfect match with the *S_11_-RNase* of *C. reticulata* (MN652907.1), while the second PCR band of 260-bp showed a match with the *S_7_-RNase* of *C. maxima* (MN652903.1). To further confirm these data, specific primers were designed on the hypervariable domains of the *S_7_* and *S_11_* (**Supplementary Table 1**; **Supplementary Figures 1, 2A**). PCR amplification led to the identification of one band for each sample (**Supplementary Figure 2B**) that was again sequenced and compared with those already identified in citrus (**Supplementary Figure 1**). The primer pairs S7FW and S7RV resulted in a fragment of 368-bp and was characterized by a 100% identity with the *S_7_-RNase* (MN652903.1) while the other primer pairs (S11FW and S11RV) amplified a fragment of 227-bp corresponding to the S11-RNase of *C. reticulata* (MN652907.1) (**Supplementary Figures 1A, B**). When comparing the *S_7_-RNase* sequence of MonSC with that of *C. maxima* (MN652903.1) a non-synonymous mutation (T>C) was detected at the nucleotide position 131 leading to the synthesis of a valine (GTT) instead of alanine (GCT) at amino acid position 44 (**Supplementary Figure 3**). This change is located between C1 and C2 in the HV1, where both amino acids are characterized by a hydrophobic side chain (**Supplementary Figure 3**). Another mutation (T>C) was identified in the *S_7_-RNase* coding sequence of MonSC at the nucleotide position 123 resulting in a synonymous amino acid mutation (**Supplementary Figure 3**). While no mutation has been found comparing the *S_11_-RNase* sequence of MonSC with that of *C. reticulata* (MN652907.1).

### 3.2 Identification of genomic variation between ComSI and MonSC using ‘Monreal’ clementine sequencing and genome assembly

The assembly of the MonSC genome resulted in a genome size of 374.2 Mb, with an N50 of 139-Kb and a mean genome coverage of 57X (**Table 1**). The quality of the *de novo* assembly was assessed using BUSCO software (**Supplementary Table 2**). Among the 1,614 genes tested, 98.1% were detected either in single (1,461; 90.5%) or in double copies (123; 7.6%), while the remaining 30 were fragmented genes (21; 1.3%) or were not detected (9; 0.6%). To further confirm the *S-RNase* identity and to retrieve the complete sequences of the two *S*-alleles, the sequences of the two amplicons were aligned against the MonSC genome. Both *S_7_-RNase* and *S_11_-RNase* were identified, confirming that the two samples shared an *S_7_S_11_*-genotype at the *S-locus*. The complete sequences of both *S_7_-RNase* and *S_11_-RNase* were then retrieved using the ‘Monreal’ reference genome, with *S_7_-RNase* located in contig 25,338, and *S_11_-RNase* in contig 4,474. The analysis of the *S_7_*- and *S_11_-RNase* sequence gene on JBrowse genome viewer confirms the presence of a single intron, that was predicted to be located inside the HV1 region (**Supplementary Figure 2A**) (Liang et al., 2020). No difference has been found in the *S_7_*- and *S_11_-RNase* sequence genes by comparing MonSC and *C. clementina* v.1 reference genomes. Similarly, no mutation has been found in the upstream and downstream regions of both *S_7_*- and *S_11_-RNases*. Also, the 1.5-kb upstream region of *S_7_-RNases*, corresponding to the promoter, was additionally analyzed for both MonSC and ComSI by Sanger sequencing, confirming the absence of mutations.

**Table 1:** Monreal genome assembly statistics.

Genomic features	‘Monreal’ clementine
Total assembly size of contigs (bp)	374,163,419
Total number of contigs	22,436
Mean size of contigs (bp)	16,676.9
Longest sequence of contigs	3,492,348
N50 length of contigs (> 150 bp)	139,000

N50, sequence length of the shortest contig at 50% of the total genome length.

To identify causative mutations related to the loss of SI phenotype in MonSC, the DNA reads of MonSC were aligned against the *C. clementina* v.1 reference genome and the resulting VCF file was filtered using the SnpEff software to retain only variants affecting the gene expression. To this extent, a total of 7,781 SNPs affecting 5,661 genes were detected by comparing MonSC and *C. clementina* reference genomes (**Supplementary Figure 4**). Among them, 1,615, 1688 and 2,358 genes are predicted to be affected by high-, low- and moderate-effect SNPs (**Supplementary Figure 4**; **Supplementary Table 3**). The distribution of the SNPs in MonSC has been visualized along the 9 chromosomes showing the highest number of SNPs in the scaffold 3 (**Supplementary Figure 5**; **Supplementary Table 3**).

### 3.3 Transcriptome analysis

**Figure 1:**
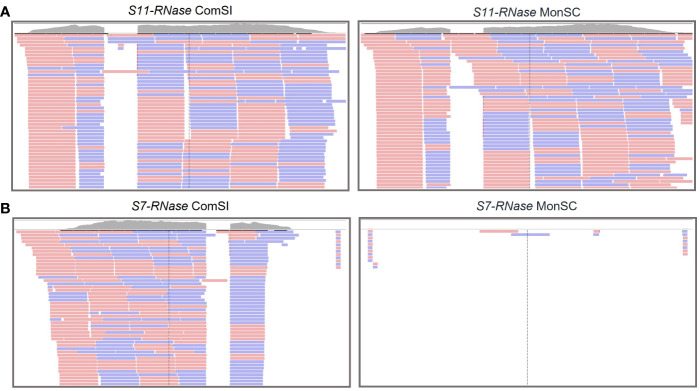
Integrative Genomics Viewer (IGV) tracks displaying sequencing read clusters of *S_11_-RNase* **(A)** and *S_7_-RNase* **(B)** genes from RNA-seq data generated from the styles and stigma of ComSI and MonSC. The grey bars depict the number of the reads mapped to the reference. Alignment of the RNA mapping is shown below in pink and blue, representing the different read strands.

RNA-seq analysis was performed on style and stigma tissues sampled 24 h after the anthesis of MonSC and ComSI to validate the role of the *S*-alleles in the SI system and to further investigate the involvement of other regulation mechanisms. Both samples expressed the *S_11_-RNase* gene (**Figure 1A**); while for the *S_7_-RNase*, the gene was not expressed in MonSC, the number of mapped reads in ComSI was comparable to what observed for *S_11_-RNase* (**Figure 1B**).

**Figure 2:**
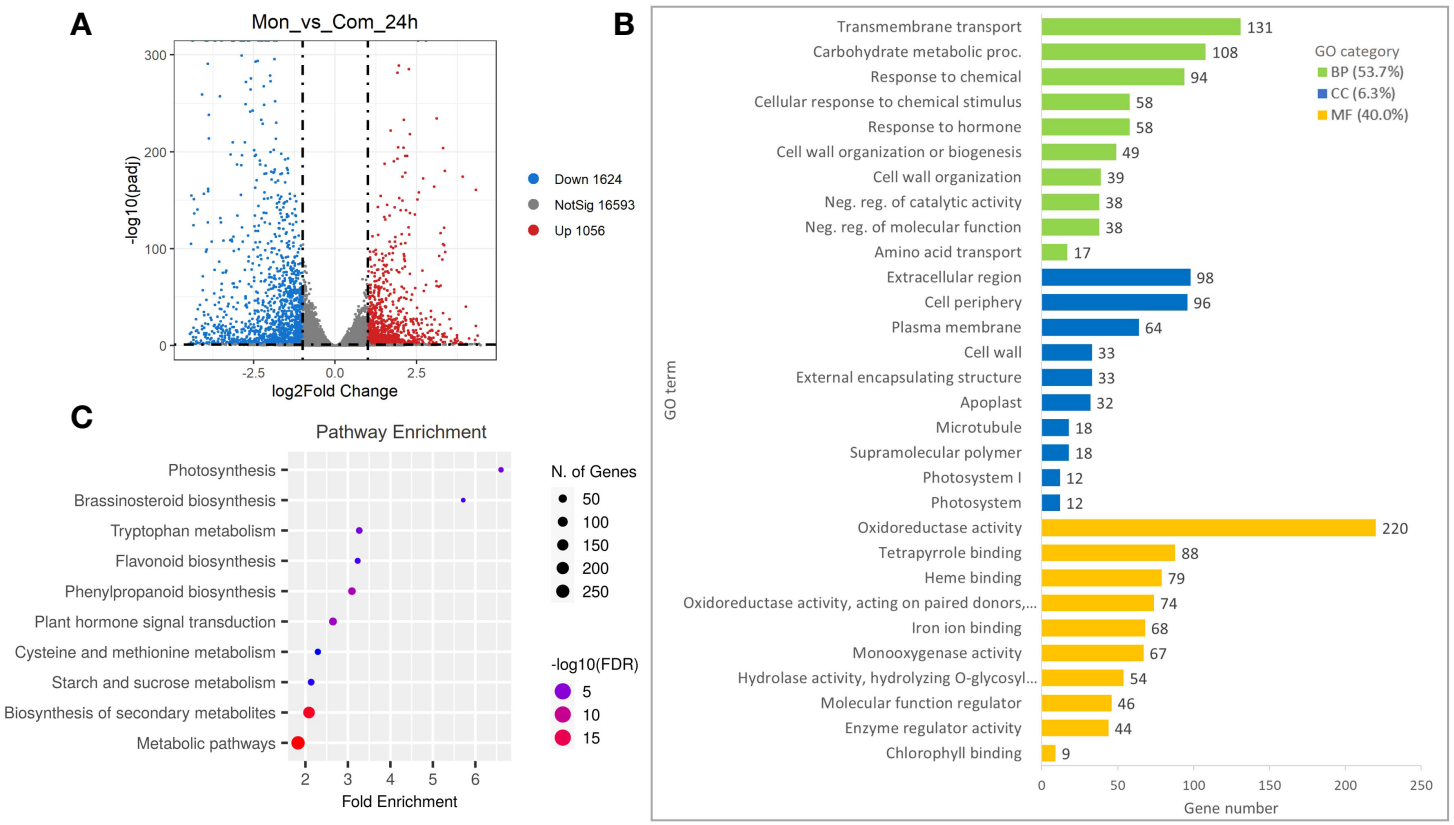
Differentially expressed genes (DEGs) in style tissues from MonSC and ComSI at 24h after anthesis. **(A)** Volcano plot of DEGs determined by RNA-seq using the criteria *p*-value ≤ 0.01 and |log2 (Fold Change)| ≥ 1. Red dots indicate up-regulated genes and blue dots indicate down-regulated genes. **(B)** Top 10 enriched GO terms in the “molecular function” (MF), “cellular component (CC) and “biological processes” (BP) categories of DEGs; **(C)** Top 10 significantly enriched KEGG (FDR ≤ 0.05) pathways in the 2,680 DEGs.

To further investigate the occurrence of genes responsible for the regulation of the *S_7_-RNase*, an analysis of the DEGs detected at a FoldChange ≥ 2 and *p*-value ≤ 0.01 was carried out. Compared with ComSI, a total of 2,680 DEGs were detected in MonSC, of which 1,056 were up-regulated and 1,624 were down-regulated (**Figure 2A**; **Supplementary Table 4**). To understand the main biological functions associated with the DEGs, a Gene Ontology (GO) enrichment analysis was performed resulting in the identification of 255 GO terms, the 53.7% belong to biological processes (BP: *n* = 137), the 6.3% to cellular components (CC: *n* = 16), and the 40% to molecular functions (MF: *n* = 100) (**Figure 2B**; **Supplementary Table 5**). The main biological functions in the BP category were ‘Transmembrane transport’ (GO:0055085, 131 genes), ‘Carbohydrate metabolic process’ (GO:0005975, 108 genes), and ‘Response to chemical’ (GO:0042221, 94 genes). In the CC category, the top three terms were ‘Extracellular region’ (GO:0005576, 98 genes), ‘Cell periphery’ (GO:0071944, 96 genes), and ‘Plasma membrane’ (GO:0005886, 64 genes). In the MF category, the top three terms were ‘Oxidoreductase activity’ (GO:0016491, 220 genes), ‘Tetrapyrrole binding’ (GO:0046906, 88 genes), and ‘Heme binding’ (GO:0020037, 79 genes) (**Figure 2B**). To gain insight into the metabolic pathways associated with DEGs, a KEGG pathway enrichment analysis was carried out (**Supplementary Table 6**). In total, 631 DEGs were categorized into 14 KEGG pathways showing FDR ≤ 0.05. The main enriched pathways were the metabolic pathways (cic01100, 257 genes) and the biosynthesis of secondary metabolites (cic01110, 167 genes) (**Figure 2C**).

### 3.4 Candidate genes involved in loss of SI in MonSC based on SNPs, DEGs and *S-RNases* promoter analysis

To identify candidate genes involved in loss of SI in MonSC the results of DNA and RNA sequencing have been combined. Among the total of 7,781 SNPs detected by aligning MonSC reads against the *C. clementina* v.1 reference genome, 807 appear to affect 650 of the 2,680 DEGs (**Supplementary Figure 4**; **Supplementary Table 3**). The candidate genes regulating *S-RNases* expression and involved in the loss of SI in MonSC were selected based on the occurrence of a SNP with high effect and on the predicted gene function (associated with flowering or SI) (*n.* 164, **Supplementary Figure 4**; **Supplementary Table 3**). This allowed us to select *CICLE_v10023354mg, CICLE_v10006615mg* and *CICLE_v10015069mg* genes encoding two members of the *Agamous*-like MADS-box family of transcription factors (*AGL12* and *AGL61*) and one belonging to the *Mildew locus O* (*MLO*-like protein 12) gene families, respectively (**Supplementary Table 3**). Also, the PlantCARE analysis of the 2.0-kb upstream sequences of *S_7_*- and *S_11_-RNase* genes showed binding sites for several classes of genes. Among them, *CICLE_v10005376mg*, encoding for a *MYB111* gene, was characterized by both a significant differential expression between the two genotypes and the occurrence of a SNP with high effect. In particular, eight binding sites were specific for the *S_7_-RNase*, while only two were specific for *S_11_-RNase* (**Supplementary Table 7**).

### 3.5 Validation of genes involved in the loss of SI by qRT-PCR

**Figure 3:**
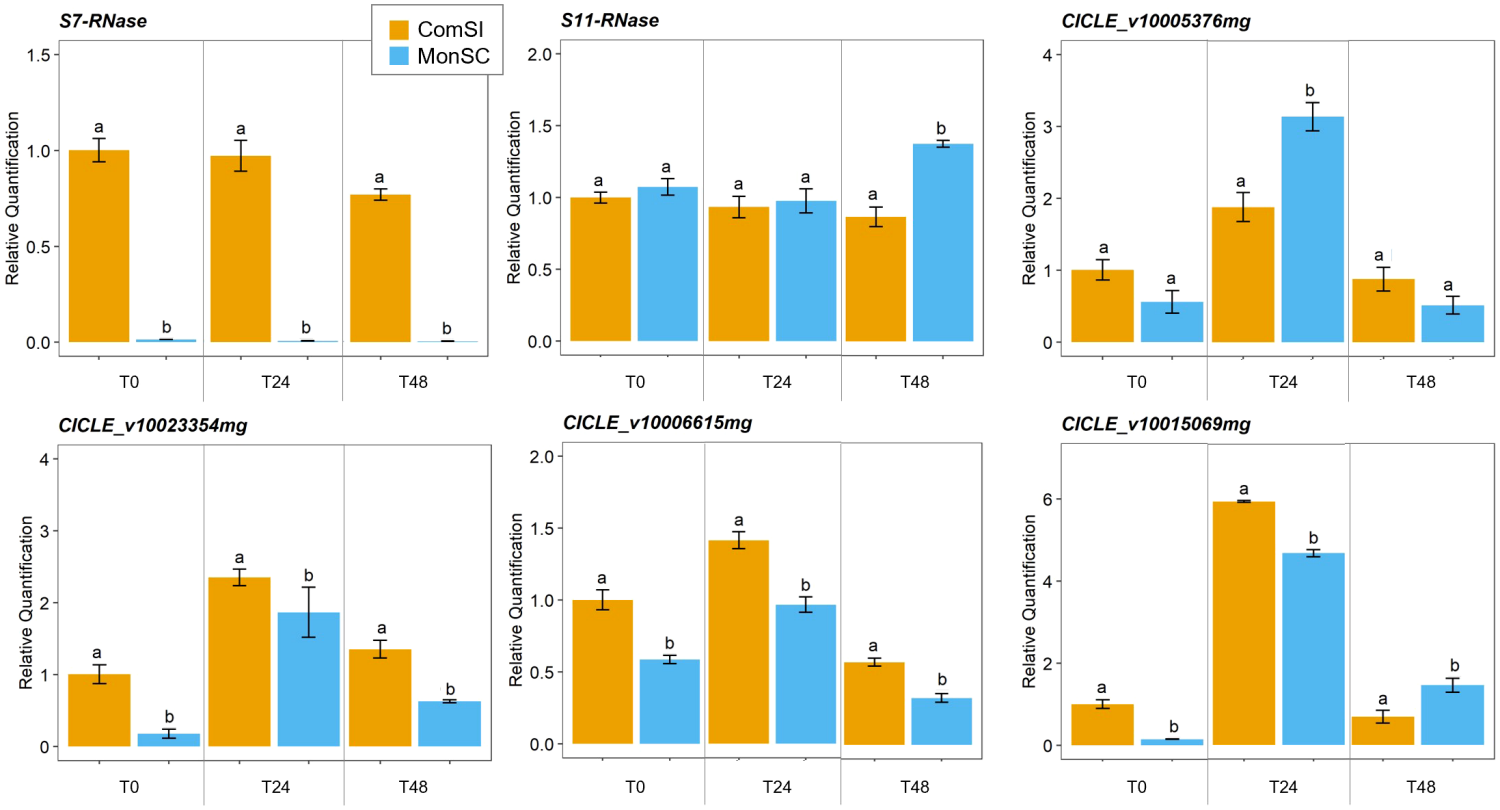
Relative quantification of expression of *S_7_-* and *S_11_-RNases* and the four selected candidate genes *CICLE_v10023354mg* (*Agamous*-like MADS-box protein AGL12), *CICLE_v10006615mg* (*Agamous*-like MADS-box protein AGL12), *CICLE_v10015069mg* (*MLO*-like protein 12) and *CICLE_v10005376mg* (transcription factor MYB111) at anthesis (COM_0 and MON_0), 24h (COM_24 and MON_24) and 48h (COM_48 and MON_48) after anthesis. Data are presented as the mean relative expression ± SD of each individual sample. Statistical analyses were performed using a t test between the gene expression level of the two genotypes at the same time-point; letters above the bars indicate significantly different values at *p*-value < 0.05).

The expression of the *S_7_-* and *S_11_-RNase* genes and four candidate genes (*CICLE_v10023354mg, CICLE_v10006615mg, CICLE_v10015069mg* and *CICLE_v10005376mg*) was further validated though a qRT-PCR assay (**Supplementary Table 1**). The expression patterns of the six candidate genes were evaluated in virgin style and stigma tissues at three stages: at anthesis, 24h and 48h after anthesis. The expression levels of the *S_7_-RNase* gene were significantly downregulated in the MonSC compared to ComSI during all the three stages while no significant differences were observed in the expression of the *S_11_-RNase* gene between MonSC and ComSI style and stigma tissues (**Figure 3**). A significant high expression of *S_11_-RNase* was observed for MonSC compared to ComSI at 48h after anthesis (**Figure 3**). Whereas the expression patterns of the four candidate genes, all showed the highest expression levels at 24h after anthesis. Among them, *CICLE_v10005376mg* (*MYB111*) showed a significantly higher expression level in MonSC with respect to ComSI, while *CICLE_v10023354mg* (*AGL12*), *CICLE_v10006615mg* (*AGL61*), and *CICLE_v10015069mg* (*MLO*-like protein 12) showed a significantly reduced expression level in MonSC compared to ComSI. Overall, the results are consistent with the RNA-seq analysis.

## 4 Discussion

The presence of a GSI system in citrus has been recently confirmed by the discovery of several *S-RNases* in pummelo that inhibit pollen tube growth (Liang et al., 2020). The identification of other *S-RNases* together with studies combining transcriptomic, phylogenetic and genetic approaches studying *S-RNase* segregation (Ren et al., 2020; Honsho et al., 2021; Ollitrault et al., 2021) offers new insights on the citrus GSI mechanism.

In this study we first characterized the *S*-genotype of clementine ComSI and MonSC that shared an *S_7_S_11_*-genotype; being the clementine reference genome haploid, the entire *S*-locus was already mapped, but only *S_11_-Rnase* together with *S_11_-SLF* genes were localized at the beginning of the pseudo-chromosome 7 (Ramanauskas and Igić, 2017; Liang et al., 2020). Here the *S_7_S_11_*-genotype is attributed for the first time to MonSC and ComSI referring to two deposited sequences, *S_7_-RNase* (MN652903.1) and *S_11_-RNase* (MN652907.1), submitted to GenBank in 2019 by Liang et al. (2020). Previous studies attributed by phenotypic observation to ComSI and ComSC an *S_3_S_11_*-genotype (Kim et al., 2020; Ollitrault et al., 2021) but, once each *S-RNase* is linked univocally to a deposited sequence, all novel studies will follow this allele numeration and previous attribution should be interpreted according to the new classification.

All known *S-RNases* belong to the Class III of T2 RNase protein family (Igic and Kohn, 2001) share several characteristics such as the *locus* architecture, the expression patterns and similar isoelectric points (Ramanauskas and Igić, 2017); all *S-RNases* contain two conserved amino acid sequences, CAS I (‘F–HGLWPV’) and CAS II (‘FW—W–HGS’), located respectively on or near C2 and C3 domains; they include two histidine residues, His46 and His109, that are essential for the ribonuclease activity (Kawata et al., 1990; Parry et al., 1997; Honsho et al., 2021). The *S_7_-RNase* and *S_11_-RNase* identified in this study shared these features; the only difference is found in the deduced amino acid sequence of MonSC S_7_-RNase. The substitution involved two amino acids, both having a hydrophobic side chain and occurring in a hypervariable region, so we can hypothesize that this variation is not affecting S_7_-RNase activity.

To decipher the molecular mechanism underling the loss of SI phenotype in MonSC, the reference genome of MonSC was presented here for the first time. The analysis of the *S_7_*- and *S_11_-RNase* genes from the MonSC genome show the absence of any mutations in their nucleotide sequence including the upstream and downstream regions. This result confirms the hypothesis that the *S_m_-RNase*, the mutated allele that was putatively attributed to the conversion of SI to SC in citrus by Liang et al. (2020), was not present in ComSI, suggesting that the occurrence of SC in MonSC is an independent event.

SI was already studied by comparing SI genotypes with their natural SC mutants. Hu et al. (2021) characterized the *S*-genotype of two pummelos (‘Shatian’ and ‘Guiyou No.1’). Both pummelos had an *S_1_S_2_*-genotype at the *S-locus*, but the *S_2_-RNase* was not expressed in the SC mutant ‘Guiyou No.1’; the reason for the lack of *S_2_-RNase* expression was not clarified even though neither structural variants, nor different level of methylation of cytosine in the two *S-RNases* were detected (Hu et al., 2021). Honsho et al. (2021), described similar results after an RNA-seq analysis of the cultivar Hyuganatsu (SI) and its natural SC mutant. The transcriptomic analysis revealed that one of the *S-RNases* (*S_15_-RNase*) was down-regulated in the SC genotype. In the two works, the SC trait seems to be associated with the downregulation of one of the two *S-RNases*, even though the mechanism responsible for the gene silencing was not clearly understood. Similarly, the *S_m_-RNase* from *C. sinensis* showed a low level of expression in the SC cultivar with respect to the SI counterpart (Liang et al., 2020).

In the present work, RNA-seq and qRT-PCR analysis revealed the lack of expression of *S_7_-RNase* in MonSC to be putatively involved in the loss of SI (**Figures 1**, **3**). As for *S_11_-RNase* a significant high expression was detected in MonSC compared to ComSI 48h after anthesis. This difference is likely not affecting the SI mechanism suggesting partial SI.

Previous studies on ComSI and MonSC have shown that the mutation affected the pistil (Distefano et al., 2009b; Caruso et al., 2012; Aloisi et al., 2020). Transcriptome comparison between pistils from ComSI and MonSC found that a total of 2,680 DEGs were present in MonSC (**Figure 2A**); among these, 631 DEGs were categorized into 14 KEGG pathways and include metabolic biosynthesis of secondary metabolites and plant hormone signal transduction, suggesting that oxidoreductase activity and metabolic pathways may participate in the regulation of *S_7_-RNase* expression; in our study, ‘Oxidoreductase activity’, ‘Transmembrane transporter activity’ and ‘Transporter activity’ were the top three terms present in the category of molecular function. These data agree with those found by Zhang et al. (2015) who performed an RNA-seq analysis on ‘Xiangshui’ SI seedless lemon and found that catalytic, transporter activity and binding were the main molecular functions present, occurring in the cell, in the membrane and in organelle. Subtractive hybridization libraries with cDNA microarray were employed to study the molecular mechanism involved in ‘Wuzishatangju’ (*C. reticulata* Blanco) a SI mandarin (2015; Miao et al., 2013). Results highlighted the involvement of genes that act by regulating signaling pathways, but also other processes like pollen development, receptor kinases, ubiquitination, calcium ion binding, gibberellin stimulus, and transcription regulation (2015; Miao et al., 2013). The involvement of a signaling cascade with reactive oxygen species (ROS) in the SI system has already been demonstrated in Chinese cabbage (*Brassica rapa* L. ssp. *pekinensis*) one of the *Brassicaceae* that, in contrast to *Citrus*, showed a SSI; in this species, self-pollen is rejected at the stigma level by the presence of high level of ROS present at the contact site of self-pollen grain that could immediately cause its arrest in SI genotype. Specific receptors, NADPH oxidases, respiratory burst oxidase homologous and GTPase regulate the level and the transport of ROS inside and between the cells (Zhang et al., 2021). Our transcriptomic result suggests that oxidoreductase activity could participate also in the regulation of SI in citrus; recently a polyamine oxidase 2 (*CrPAO2*), responsible for spermine and spermidine oxidation leading to the production of H_2_O_2_, was up-regulated in pollen of ‘Wuzishatangju’ (*C. reticulata* Blanco) a SI mandarin in respect to the SC mutant (Ren et al., 2020).

In order to detect candidate genes regulating *S-RNase* expression and SI/SC mechanisms genomic and transcriptomic data were combined. A total of 7,781 SNPs affecting 5,661 genes were detected by comparing MonSC and *C. clementina* reference genomes (**Supplementary Figure 4**) of which 164 DEGs were characterized by the presence of at least one high-effect SNPs (**Supplementary Figure 4**; **Supplementary Table 3**). Some of the identified genes include several transcription factors and genes associated with flowering such as *Agamous*-like MADS-box and *MLO*-like protein 12. *AGAMOUS-LIKE 61* (*AGL61, CICLE_v10006615mg*) also termed *DIANA* or *DIA*, is a type I MADS box involved in embryo formation and seed growth, in Arabidopsis; loss of *AGL61/DIA* function impairs central cell maturation and shows decreased female fertility (Bemer et al., 2008; Steffen et al., 2008). *AGAMOUS-LIKE 12* (*AGL12, CICLE_v10023354mg*) also termed *XAANTAL1* (*XAL1*) encodes for a transcription factor that belong to type II Arabidopsis MADS-box genes; it is implicated in root development and flowering transition, in particular this gene participates in the photoperiod pathway (Tapia-López et al., 2008; Rodríguez-Bolaños et al., 2023). The *MLO*-like protein 12 belongs to *MLO* genes that encode plasma membrane proteins with a calmodulin-binding domain essential for successful colonization of powdery mildew pathogens in numerous crops (Devoto et al., 1999; Kim et al., 2002). *MYB111* appeared to contain multiple binding sites in the 2.0-kb upstream sequences that were univocal for *S_7_*- or *S_11_-RNase* genes (**Supplementary Table 7**). *MYB111* is a member of the R2R3-MYB transcription factor family, the largest subclade of plant MYB transcription factors (Stracke et al., 2007) and it has been shown to promote and regulate the synthesis of flavonoids; in Arabidopsis *MYB111* can bind to specific cis-elements in promoter of chalcone synthase (*CHS*), flavanone carboxylase (*F3H*), flavonol synthase 1 (*FLS1*), and in turn activates their transcription (Stracke et al., 2007; Li et al., 2019). Flavonoids are considered plant stress moderators, they protect plants when exposed to diverse environmental stresses including salinity, drought and UV radiation and having strong antioxidant properties, and can scavenge reactive oxygen species (ROS) stimulating the defense system (Shomali et al., 2022).

The different expression of the *S*-allele in the SC genotype with respect to the SI genotype has also been detected in other species. In almond (*Prunus amygdalus* Batsch) the presence of epigenetic changes in several cytosine residues were detected in the 5’ upstream region of SC samples (Fernández i Martí et al., 2014), while no difference was found in the coding or regulatory sequences of both SC and SI alleles nor in the whole chromosome region bordering the *S*-locus except for the differentially expressed *S-RNase* (Fernández i Martí et al., 2010); whole genome bisulfite sequencing of SC and SI pummelo cultivar resulted in no significant variation of the methylated cytosine (Hu et al., 2021).

## 5 Conclusion

In the presented work, the genetic mechanism of SI has been investigated in the SI ‘Comune’ clementine (ComSI) and in its SC mutant ‘Monreal’ (MonSC). PCR analysis allowed the characterization of the complete *S*-genotype of the samples, both showing an *S_7_S_11_*-genotype. *S_7_-RNase* in particular is here presented for the first time since the reference genome of *C. clementina* reported only one *S*-allele (*S_11_*). A high-quality reference genome of ‘Monreal’ clementine was employed to assess the mutation rate between MonSC and the *C. clementina* reference genome. Pistil RNA-seq analysis highlighted the lack of expression of *S_7_-RNase* in MonSC and gene ontology identified the highest fraction of DEGs among the oxidoreductase and transmembrane transport activity groups. To dissect the genetic regulation of SI, transcriptomic and genomic data were combined allowing the identification of 164 DEGs characterized by the presence of at least one high-effect SNPs. Four candidate genes associated with flowering and referring to two *Agamous*-like MADS-box proteins, to *MLO*-like protein 12 and to *MYB111*, were selected suggesting their putative involvement in the regulation of SI. Among them, the transcription factor *MYB111* appeared to contain several binding sites in the promoter region of *S_7_*- or *S_11_*-*RNase* genes. The identification of such genes paves the way for additional analyses aimed at functionally elucidate their role and validate their involvement in the SI process. This study provides novel molecular tools that researchers can use to better clarify the physiological regulation of SI. Additionally, breeders can utilize these tools to establish innovative breeding programs aimed at the development of improved seedless varieties.

## Data availability statement

The datasets presented in this study can be found in online repositories. The names of the repository/repositories and accession number(s) can be found below: NCBI repository, accession number PRJNA1053323, PRJEB72497.

## Author contributions

SB: Writing – original draft, Investigation, Formal analysis. LP: Writing – original draft, Investigation. MDG: Writing – original draft, Formal analysis, Data curation. LP-A: Writing – review & editing, Data curation. MC: Writing – review & editing. CL: Writing – review & editing. AG: Writing – review & editing, Funding acquisition. GD: Writing – review & editing, Conceptualization. SLM: Writing – review & editing, Funding acquisition.
