# Supplementary material for: The origin and the genetic regulation of the self-compatibility mechanism in clementine (Citrus clementina Hort. ex Tan.)
Source: Front Plant Sci. 2024 Mar 4;15:1360087. doi: 10.3389/fpls.2024.1360087 (PMC10944956; doi:10.3389/fpls.2024.1360087)
Supplement: Supplementary file 1 [file DataSheet_1.docx]

Supplementary Material

# Supplementary Figures


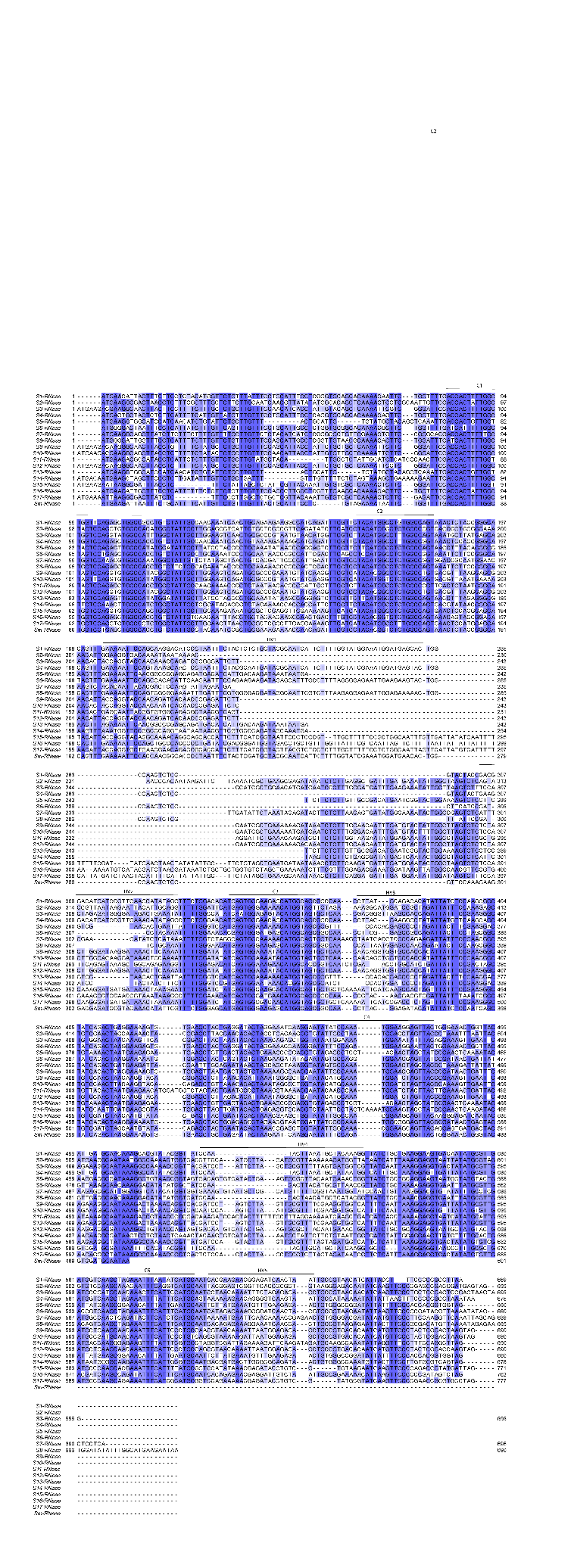


**Supplementary Figure 1**. Nucleotide sequence alignment of *S-RNases* from *C. maxima* [*S_1_-S_9_-RNases* (MN652897.1, MN652898.1, MN652899.1, MN652900.1, MN652901.1, MN652902.1, MN652903.1, MN652904.1, MN652905.1) and *S_16_-RNase* (LC575207.1), *C. reticulata* [*S_10_-S_11_-RNases* (MN652906.1 and MN652907.1), *Atalantia buxifolia* [*S_12_-RNase* (MN652908.1)], *C. cavaleriei* [*S_13_-RNase* (MN652909.1)], *C. medica* [*S_14_-RNase* (MN652910.1)], *C. tamurana* [*S_15_-RNase* (LC575202.1)], *C. hassaku* [*S_17_-RNase* (LC575209.1)], and *C. sinensis* [*S_m_-RNase* (MN652911.1)]. The alignment was generated with Clustal Omega and Jalview v.2.11.2.6. Nucleotides are color coded according to their conservancy: dark violet are fully conserved, while those marked in light violet present less similar. Five conserved domains (C1-C5) and five hypervariable regions (HV1-HV5) are indicated.


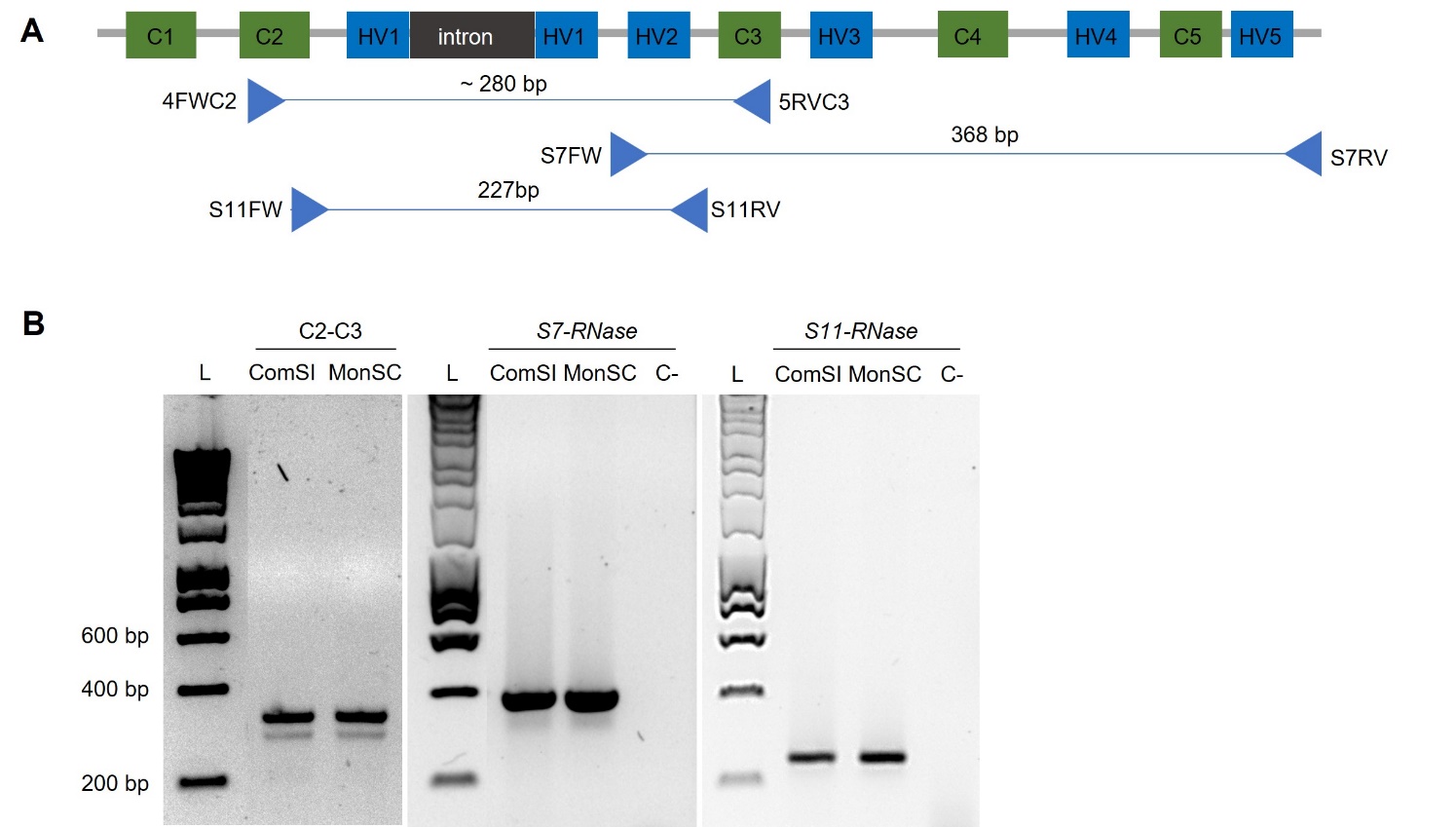


**Supplementary Figure 2**. Analysis of *S-RNases* in ComSI and MonSC. (a) Schematic representation of the *S-RNase* shows 5 conserved (C1-C5, in green) and hypervariable domains (HV1-HV5, in blue) and an intron (in grey). Arrows at the bottom indicate PCR primers used to amplify C2 and C3 domains, the *S_7_* and *S_11_* alleles and the respective size of PCR amplicons. (b) Visualization on agarose gel of amplified PCR product using degenerate (4FWC2 and 5RVC3) and specific (S7FW, S7RV, S11FW, and S11RV) primers; C- = negative controls; L = ladder.


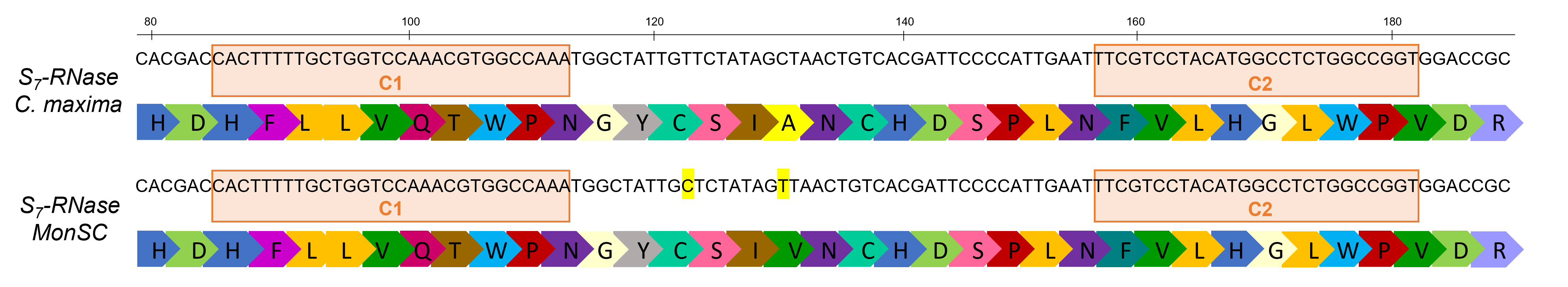


**Supplementary Figure 3**. Comparison of the nucleotide and amino acid sequences of *S7-RNase* from *C. maxima* and MonSC.


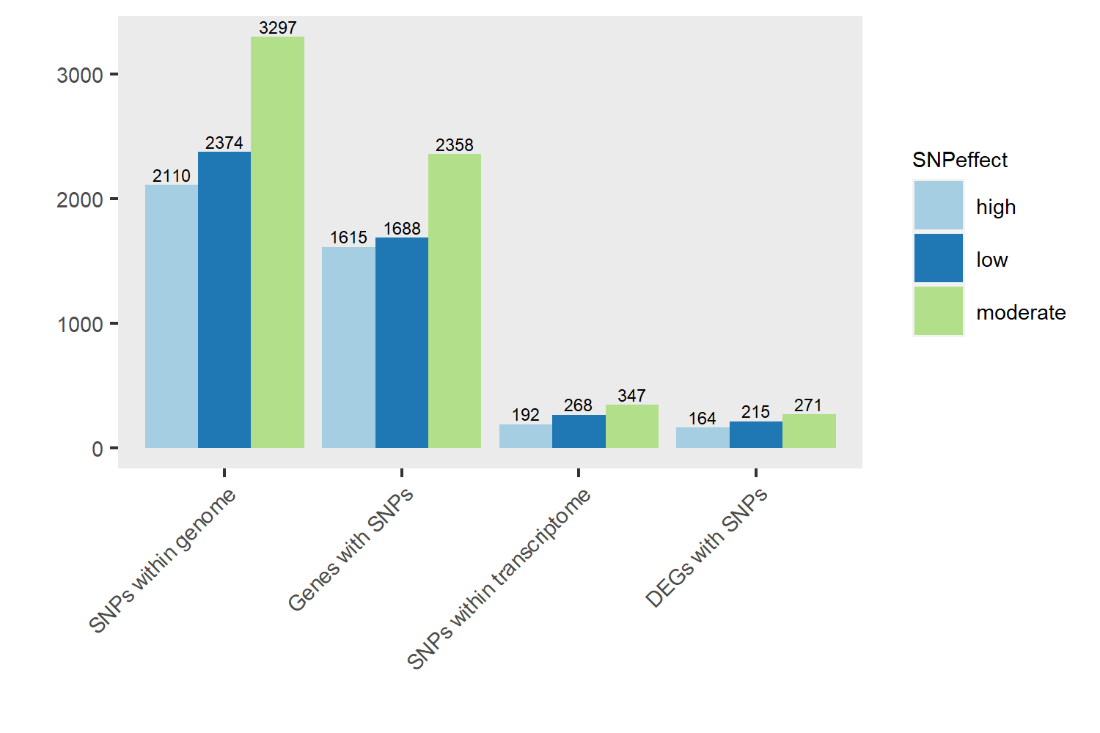


**Supplementary Figure 4**. SNPeff analysis. The number of SNPs affecting genome and transcriptome and number of genes and DEGs affected by SNPs by comparing MonCS against ComSI. SNPs are classified according to their impact in high (light blue), low (blue) and moderate (green).


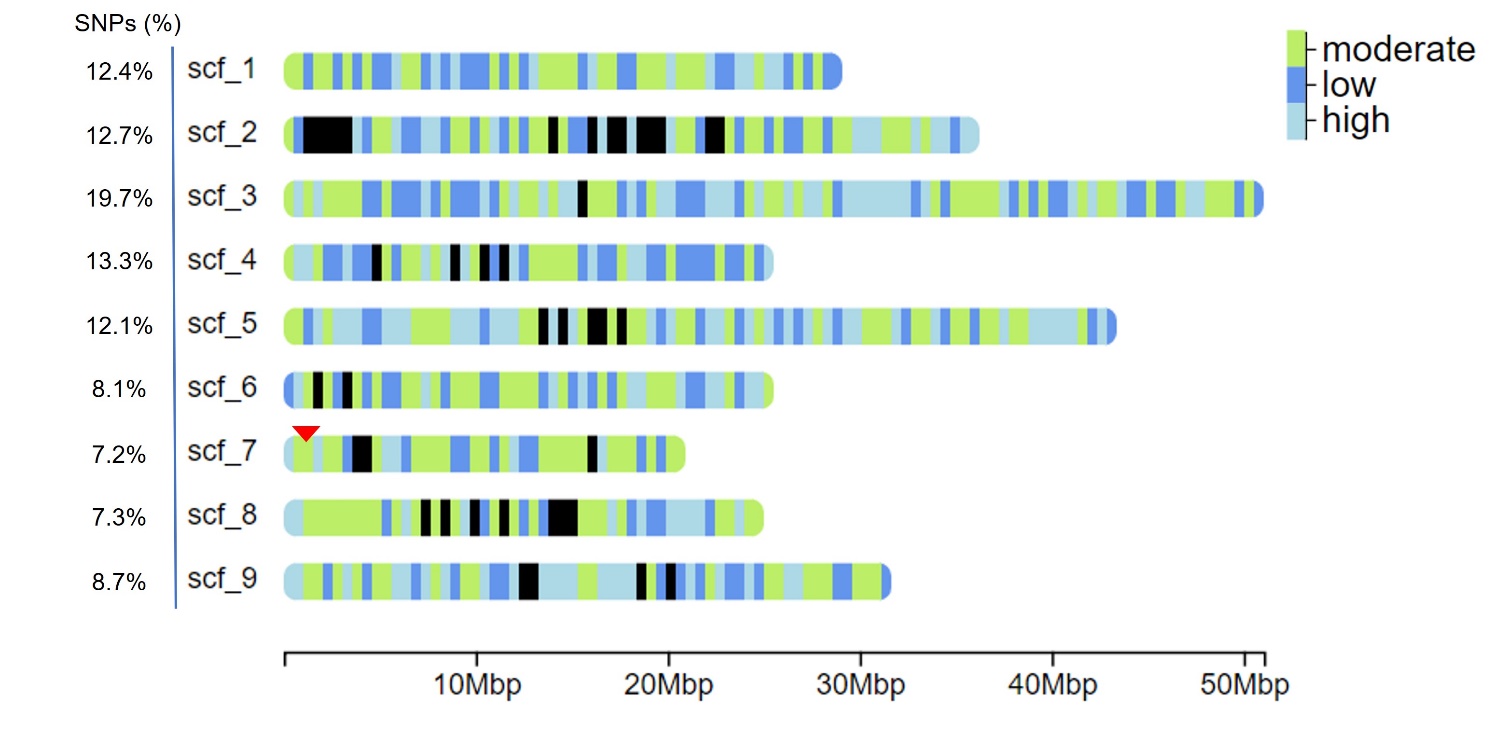


**Supplementary Figure 5**. SNPs distribution in MonSC colored according to their impact in high (light blue), low (blue) and moderate (green). At the left are indicated the percentage of SNPs along the scaffold. The red arrowhead indicates the position of the *S-RNase*.

# Supplementary Tables

**Supplementary Table 1.** List of primers designed in this study.

| **Primer name** | **Target** | **Sequence (5’-3’)** | **Product size** | **PCR** |
| --- | --- | --- | --- | --- |
| 4FWC2 | C2 conserved domain of *S-Rnase* | TWCAYGGYCTCTGGCCVGT | ~ 280 | Consensus |
| 5RVC3 | C3 conserved domain of *S-Rnase* | TRCCRTGYWBWABCCACT |  |  |
| S7FW | *C. maxima S_7_-RNase* (MN652903.1) | TGGCCGAGTCTCATTTCGAAG | 368 | Allele-specific |
| S7RV |  | TCCCCACAGTTCTCGGTTTTG |  |  |
| S11FW | *C. reticulata S_11_-RNas*e (MN652907.1) | CGCTGTTCAGAGTAAAGCTGGC | 243 |  |
| S11RV |  | AGTGGATCTTTGTCGCGGGTTA |  |  |
| PS7.1FW | MonSC contig_25338: 15,956-16,612 | TGAAGCGCGGTTCTATTTTC | 657 | *S_7_-RNase* promoter |
| PS7.1RV |  | TGAAGCGCGGTTCTATTTTC |  |  |
| PS7.2FW | MonSC contig_25338: 15,219-15,984 | AGATGATTTGCTGTTGCACG | 766 |  |
| PS7.2RV |  | TTGGGGATAATTCAACAGCC |  |  |
| S7RTFW | *C. maxima S_7_-RNase* (MN652903.1) | TTTCGTCCTACATGGCCTCTGG | 150 | qRT-PCR |
| S7RTRV |  | TTCGAAATGAGACTCGGCCAGT |  |  |
| S11RTFW | *C. reticulata S_11_-RNas*e (MN652907.1) | TCTGGCCCGTTGACTCTAATGG | 146 |  |
| S11RTRV |  | GCCAGCTTTACTCTGAACAGCG |  |  |
| AGL61FW | *CICLE_v10006615mg* (XM_006421285.1) | CTGTCTGGGAAGCCTTTCAC | 106 |  |
| AGL61RV |  | CCAGCGGATGAGTGTTATCG |  |  |
| AGL12FW | *CICLE_v10023354mg* (XM_006441530.2) | AACCATGCAAGGGCTAATTG | 151 |  |
| AGL12RV |  | CATATACCTGAGGCCTTTTTGG |  |  |
| MLOFW | *CICLE_v10015069mg* (XM_006449356.2) | TAACCCTAAGGCGTGGATTC | 228 |  |
| MLORV |  | TAGTTCCCACCACCAATAGC |  |  |
| MYBFW | *CICLE_v10005376mg* (XM_024189929.1) | GTGGTGCTGAGTGTCATAATC | 183 |  |
| MYBRV |  | TAAACCTTTGTGCATCTCAAC |  |  |

**Supplementary Table 2.** Benchmarking Universal Single-Copy Orthologs (BUSCO) analysis and descriptive statistics. Size of the genome, Number of scaffold and N50 of the MonSC genome compared with the related genome of ComSI and *C. reticulata*. In all genomes 1,614 target genes of the ‘embryophyta’ lineage were considered for the BUSCO analysis.

| **Species name** | | **‘Monreal’ clementine** | ***Citrus* x *clementine*** | ***C. reticulata*** | |
| --- | --- | --- | --- | --- | --- |
| Version | | V1.0 | V1.0 | V1.0 | |
| Complete BUSCOs | Number | 1,584 | 1,366 | 1,345 |  |
|  | % | 98.1% | 94.90% | 93.40% |  |
| Complete and single-copy BUSCOs | Number | 1,461 | 1,329 | 1,309 |  |
|  | % | 90.5% | 92.30% | 90.90% |  |
| Complete and duplicated BUSCOs | Number | 123 | 37 | 36 |  |
|  | % | 7.6% | 2.60% | 2.50% |  |
| Fragmented BUSCOs | Number | 21 | 36 | 49 |  |
|  | % | 1.3% | 2.50% | 3.40% |  |
| Missing BUSCOs | Number | 9 | 38 | 46 |  |
|  | % | 0.6% | 2.60% | 3.20% |  |
| *Total BUSCO groups searched | | 1,614** | 1,440* | 1,440* | |
| Total size of assembly (kb) | | 374,163 | 301,387 | 347,457 | |
| No. of scaffolds/contigs | | 22,436 | 1,398 | 90,139 | |
| N50 (kb) | | 139 | 31,410 | 1,585 | |

BUSCO was run in mode: proteins; Dataset: * "embryophyta_odb9"; **embryophyta_odb10

**Supplementary Table 3.** List of SNPs obtained comparing MonSC and *C. clementina* reference genome predicted to have an high, moderate or low effect on gene.

**Supplementary Table 4.** DEGs detected at a FoldChange ≥ 2 and *p*-value ≤ 0.01.

**Supplementary Table 5.** Output of Gene Ontology (GO) enrichment analysis.

**Supplementary Table 6.** KEGG pathway enrichment analysis results.

**Supplementary Table 7.** Feature of motif associated with DEGs affected by high-effect SNPs in the 2.0-kb upstream sequence of *S_7_*- and *S_11_-RNase* genes from PlantCARE database.

| **Motif** | **Sequence** | **Location** | **Strand** | **S-RNase promoters** |
| --- | --- | --- | --- | --- |
| MYB | CAACAG | 772 | - | *S_7_-RNase* |
| MYB | TAACCA | 971 | + | *S_7_-RNase* |
| MYB | CAACAG | 1192 | - | *S_7_-RNase* |
| MYB | CAACAG | 1930 | - | *S_7_-RNase* |
| MYB-like sequence | TAACCA | 971 | + | *S_7_-RNase* |
| MYB-binding site | CAACAG | 772 | - | *S_7_-RNase* |
| MYB-binding site | CAACAG | 1192 | - | *S_7_-RNase* |
| MYB-binding site | CAACAG | 1930 | - | *S_7_-RNase* |
| MYB | CAACTG | 1841 | - | *S_11_-RNase* |
| MYB | CAACCA | 487 | - | *S_11_-RNase* |
